# A Cross-Sectional Serosurvey of Anti-Orthopoxvirus Antibodies in Central and Western Africa

**DOI:** 10.3390/v9100278

**Published:** 2017-09-29

**Authors:** Siv Aina J. Leendertz, Daniel Stern, Dennis Theophil, Etile Anoh, Arsène Mossoun, Grit Schubert, Lidewij Wiersma, Chantal Akoua-Koffi, Emmanuel Couacy-Hymann, Jean-Jacques Muyembe-Tamfum, Stomy Karhemere, Maude Pauly, Livia Schrick, Fabian H. Leendertz, Andreas Nitsche

**Affiliations:** 1 Epidemiology of Highly Pathogenic Microorganisms, Robert Koch Institute, 13353 Berlin, Germany; LeendertzS@rki.de (S.A.J.L.); SchubertG@rki.de (G.S.); Lidewij.Wiersma@fao.org (L.W.); Maude.Pauly@lih.lu (M.P.); LeendertzF@rki.de (F.H.L.); 2 Department of Infectious Disease Epidemiology, Robert Koch Institute, 13353 Berlin, Germany; 3 Centre for Biological Threats and Special Pathogens ZBS 1, Highly Pathogenic Viruses Centre for Biological Threats and Special Pathogens, Robert Koch Institute, 13353 Berlin, Germany; SternD@rki.de (D.S.); Theokom@gmx.net (D.T.); SchrickL@rki.de (L.S.); 4 Université Felix Houphouët Boigny, Abidjan BP 1174, Cote D’Ivoire; anohethyl@yahoo.fr (E.A.); mossouna@yahoo.fr (A.M.); 5 Centre de Recherche pour le Développement, Université Alassane Ouattara, Bouaké BP 1174, Cote D’Ivoire; Akouamc@yahoo.fr; 6 Laboratoire National D’appui au Développement Agricole/Laboratoire Central de Pathologie Animale, Bingerville BP 206, Cote D’Ivoire; chymann@hotmail.com; 7 Institut National de Recherche Biomédicale, Kinshasa BP 1197, Democratic Republic of the Congo; jjmuyembet@gmail.com (J.-J.M.-T.); Stomy.Karhemere@inrb.cd (S.K.); 8 Department of Infection and Immunity, Luxembourg Institute of Health, 4354 Esch-sur-Alzette, Luxembourg

**Keywords:** orthopoxvirus, seroprevalence, zoonoses, ELISA (enzyme linked immunosorbent assay)

## Abstract

Since the eradication of smallpox and the subsequent discontinuation of the worldwide smallpox vaccination program, other Orthopoxviruses beside Variola virus have been increasingly representing a risk to human health. To investigate the extent of natural contact with Orthopoxviruses and possible demographic risk factors for such an exposure, we performed a cross-sectional serosurvey of anti-Orthopoxvirus IgG antibodies in West and Central Africa. To this end, people living in forest regions in Côte d’Ivoire (CIV, *n* = 737) and the Democratic Republic of the Congo (COD, *n* = 267) were assigned into groups according to their likely smallpox vaccination status. The overall prevalence of anti-Orthopoxvirus antibodies was 51% in CIV and 60% in COD. High rates of seropositivity among the vaccinated part of the population (80% in CIV; 96% COD) indicated a long-lasting post vaccination immune response. In non-vaccinated participants, seroprevalences of 19% (CIV) and 26% (COD) indicated regular contact with Orthopoxviruses. Multivariate logistic regression revealed that the antibody level in the vaccinated part of the population was higher in COD than in CIV, increased with age and was slightly higher in females than males. In the unvaccinated part of the population none of these factors influenced antibody level significantly. In conclusion, our results confirm expectedly high anti-Orthopoxvirus seroprevalences in previously smallpox-vaccinated people living in CIV and the COD but more unexpectedly imply regular contact with Orthopoxviruses both in Western and Central Africa, even in the absence of recognized outbreaks.

## 1. Introduction

The Orthopoxvirus (OPV) genus comprises several viruses pathogenic to humans, like Cowpox virus (CPXV), Vaccinia virus (VACV), Monkeypox virus (MPXV), and Variola virus (VARV) [1]. VARV is the causative agent of smallpox, a devastating infectious disease successfully eradicated about 40 years ago by a world-wide smallpox vaccination campaign [2,3]. Since smallpox vaccination was also highly protective against other human-pathogenic OPVs, its subsequent cessation has been increasing the probability of zoonotic OPV infections in humans [4,5]. In Europe, increasing numbers of documented infections with CPXV are being observed [6,7], while VACV-like viruses cause infections in South America [8] and MPXV cause infections in Africa [9]. CPXV is transmitted to humans mainly by direct contact to rodents or cats, and in most cases CPXV causes a self-limiting skin disease. VACV-like viruses infect dairy workers through direct contact to infected cattle. The infection also usually results in benign and self-limiting clinical signs, but severe and generalized disease has been observed in individual cases [10]. Particularly remarkable is the re-occurrence of monkeypox in forested areas of Western and Central Africa, with frequent reports from the Democratic Republic of the Congo (COD) [11]. MPXV are causing a disease with clinical signs almost identical to smallpox (fever, skin pathology), though causing more moderate mortality. For that reason, in the era of smallpox vaccination, MPXV was not recognized as a discrete entity until the 1970s [12]. Rodents, such as squirrels of the *Funisciurus* and *Heliociurus* genera, are thought to be the reservoir of the virus and the likely source of human disease [13,14]. Transmission into the human population is hypothesized to occur through hunting and preparation of food from infected species [15] while subsequent human-to-human transmission can occur via saliva and respiratory excretions or contact with lesion exudate and crust material [9,16,17,18]. The ecological factors that influence host distribution and viral emergence are not completely understood [11,16].

In contrast to VARV, which could be eradicated by systematic vaccination of those parts of the human population at risk, the eradication of a zoonotic virus with partially unknown reservoirs and a broad host-range is all but impossible. The vaccines used previously for the eradication are efficient but show a poor safety profile, and new vaccines are in the pipeline to be applicable for the general population, including people with immunological disorders [19]. Antiviral therapeutics are promising but so far there is no medication licensed to specifically treat poxvirus infections. Even if the remaining hurdles for compounds like ST-246 [20] and CMX-001 [21] are cleared, the global availability particularly in developing countries is questionable. Hence it is crucial to monitor human exposure to MPXV and other OPVs to understand better the ecology of these viruses and their zoonotic hosts and the dynamics of a possible re-emergence, and perhaps to predict future spill-over events to limit the number and size of OPV disease outbreaks. The surveillance of human populations that have substantial contact with rodents and other wildlife through hunting, preparation, and consumption of bush meat and that engage in agricultural activities in the forest/on the village borders therefore remains a promising approach for investigating epidemiological patterns of exposure to OPVs in endemic and non-endemic areas.

We therefore conducted OPV serosurveys in humans living in forest border areas in CIV and the COD with the intention of investigating possible differences in exposure to OPVs between West and Central Africa, to identify demographic risk factors for such an exposure and to verify post-vaccination antibody levels in people that were part of the smallpox eradication program up until 40 years ago.

## 2. Materials and Methods

### 2.1. Ethics Statement

All procedures performed in studies involving human participants were in accordance with the ethical standards of the institutional and/or national research committee and with the 1975 Helsinki declaration (http://www.wma.net/en/30publications/10policies/b3/) and its later amendments or comparable ethical standards. Human sampling was approved by the Ivorian ethics commission (Comité national d’éthique et de la recherche (CNER), permit number 101 10/MSHP/CNER/P) and the Congolese ethics commission (Comité d’Éthique, Ministère de l’Enseignement Supérieur et Universitaire, permit number ESO/CE/018/11, 15 June 2011). Informed consent was obtained from all participants included in the study. The questionnaire data were treated anonymously.

### 2.2. Study Populations and Sample Collection

**Figure 1:**
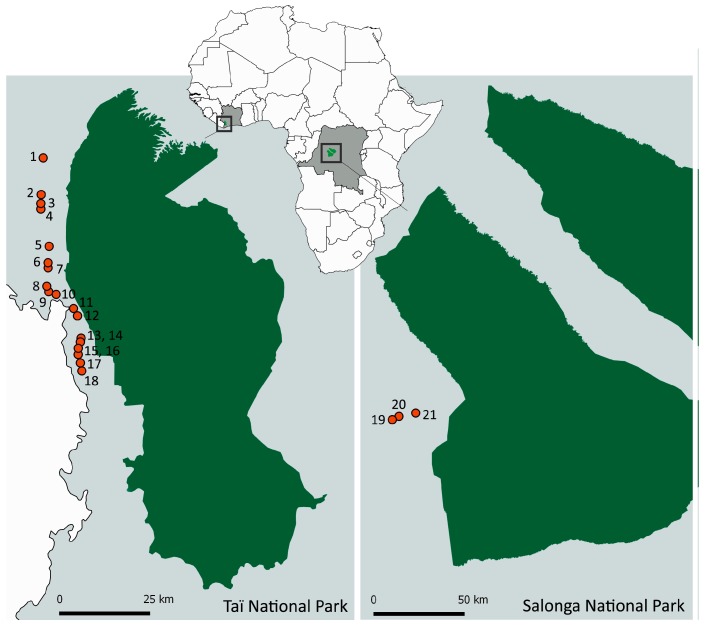
Rural sampling locations near Taï National Park, Côte d’Ivoire (left panel) and Salonga National Park, Democratic Republic of the Congo (right panel). National Parks in dark green. 1, Zagne (Zag). 2, Tienkoula (Tie). 3, Goulegui Beoue (GouB). 4, Djiboubaye (Dji). 5, Keibly (Kei). 6, Zaipobly (Zai). 7, Gahably (Gah). 8, Daobly (Dao). 9, Ponan (Pon). 10, Taï (Tai). 11, Gouleako (Gou). 12, Pauleoula (Pau). 13, Portgentil (Por). 14, Djereoula (Dje). 15, Tienkoula (Tien). 16, Tieleoula (Tiel). 17, Ziriglo (Zir). 18, Sakre (Sak). 19, Bekombo (Bek). 20, Lompole (Lom). 21, Ipope/Nganda (Ipo/Nga).

We collected blood samples from participants living in villages bordering tropical rainforests in CIV and the COD (Figure 1). The study populations are predominantly living on agriculture but also engage in forest-related activities such as hunting (rodents, primates, and other wild life) and fishing as well as a range of other village-related occupations. Volunteer participants of all ages were recruited from 18 villages in CIV (Daobly, Djereoula, Djiboubaye, Gahably, Gouleako, Goulegui Beoue, Keibly, Pauleoula, Ponan, Portgentil, Sakre, Siobloula, Tai, Tieleoula, Tienkoula, Zagne, Zaipobly, and Ziriglo) in 2006/2007 and four villages in COD (Bekombo, Ipope, Lompole, and Nganda) in 2011/2012. Three villages in CIV (Gouleako, Ponan, and Tai) were sampled also in 2012. Age and gender were recorded for each participant. The age value for babies under one year of age was set to one.

Blood was collected by venepuncture into ethylenediaminetetraacetic acid (EDTA) tubes by medical personnel from the respective countries. After centrifugation, serum was transferred into cryotubes and stored frozen until laboratory analyses were done at the Robert Koch Institute in Berlin, Germany.

All participants of the study were offered a basic clinical examination by a trained medical professional and, when necessary, free treatment and medical advice were provided. If treatment was not possible on site, individuals were referred to an appropriate medical facility.

### 2.3. Definition of the Smallpox Vaccination Status

Participants born up to 1975 (five years before the smallpox eradication declaration in 1980) were considered vaccinated, participants born between 1975 and 1985 (five years before and after the declaration of eradication) were considered to have uncertain vaccination status and participants born after 1985 (five years or more after the declaration of eradication) were considered not vaccinated.

### 2.4. Enzyme Linked Immunosorbent Assay (ELISA)

Serological tests were done using an in-house enzyme linked immunosorbent assay (ELISA) which was cross-validated against a neutralization assay and an immunofluorescence assay (IFA).

#### 2.4.1. General Procedure

To establish the in-house ELISA, Hep-2 cells (American Type Culture Collection (ATCC), Manassas, VA, USA, CCL-23™) or VACV (NYCBOH; ATCC VR-1536™)-infected Hep-2 cells (multiplicity of infection of 0.2 for 3 to 4 days until all cells detached due to cytopathic effect) were lysed with RIPA buffer (Thermo Fisher Scientific, Dreieich, Germany; supplemented with HALT™ Protease Inhibitor Cocktail), and protein content was quantified using a bicinchoninic acid assay (BCA) (Pierce, Dallas, TX, USA) according to manufacturers’ recommendations. Polysorb™ 96-well microtiter plates (Nunc, Langenselbold, Germany) were coated with 100 μL of cell lysate (4 μg/mL) in 0.1 M carbonate buffer (pH 9.6) at 4 °C overnight. To account for potential unspecific binding of serum antibodies to cellular proteins, half of the wells on each plate were coated with lysate of non-infected cells, whereas the remainder was coated with lysate of VACV-infected cells. The next day, coated plates were washed four times with 300 μL/well of washing buffer (100 mM Tris, 0.9% NaCl, pH 7.5, 0.05% Tween 20) and blocked for 1 h at 37 °C with 200 μL/well of washing buffer supplemented with 3% bovine serum albumin (bovine serum albumin) BSA; Carl Roth, Karlsruhe, Germany). Sera were diluted 1:100 in washing buffer supplemented with 0.25% BSA. After plate washing, 50 μL of diluted sera per well were incubated on VACV-infected and non-infected cell lysate for 1 h at 37 °C. After washing, bound antibodies were detected with 100 μL/well of 1:5000 diluted HRP-labeled goat anti-human IgG-specific antibodies (Invitrogen™, Thermo Fisher Scientific). Finally, the enzymatic colorimetric reaction was performed for 15 min with TMB substrate (Sigma-Aldrich, Taufkirchen, Germany), stopped by adding 1 M sulfuric acid, and analyzed by measuring the optical density at 450 nm with an Infinite200 PRO microplate reader (Tecan, Maennedorf, Switzerland).

#### 2.4.2. ELISA Data Processing and Normalization

On each ELISA plate a panel of five OPV-negative sera (immunofluorescence assay) IFA titer < 1:20), two weakly positive sera (IFA titers 1:320) and highly positive (IFA titer 1:10,240) vaccinia immune globulin (VIG, NR-2632, diluted 1:500; obtained through BEI Resources) was included as an internal quality control and for data normalization. To this end, first the difference between ELISA readings of infected and non-infected cell lysate was calculated for each serum. Next, the mean plus three times the standard deviation of the differences for the five OPV-negative sera was subtracted from difference values of test sera. Finally, all values were normalized to results obtained for VIG to minimize plate-to-plate variation (termed antibody level).

#### 2.4.3. Cross Validation by Method Comparison

For cross validation, IFA titers for a panel of 100 sera from German blood donors were determined as described previously [22]. Additionally, the amount of VACV-neutralizing antibodies was measured for a subpanel of 50 sera by a previously developed neutralization assay [23]. Correlations between both assays and the ELISA were calculated by either parametric Pearson correlation (between antibody level and cell index for the neutralization assay) or non-parametric Spearman correlation (between antibody level and IFA titer for IFA) using Prism 5.04 (GraphPad, La Jolla, CA, USA).

#### 2.4.4. Determination of the Seroprevalence

For prevalence estimations, an antibody level cut-off of 0.05 was used to determine if the sample was positive or negative. For overall prevalence in the respective countries, all samples irrespective of vaccination status were included. A 95% confidence interval was calculated for all prevalence estimates.

### 2.5. Stastistical Analyses

To test whether antibody levels were influenced by age, gender, and/or country, we used a general linear mixed model [24] into which we simultaneously included these three predictors as fixed effects. To control for village, this was added as random effect. Data from samples collected in the main sampling seasons from CIV 2006/2007 and COD 2011/2012 was included, and models were run separately for the populations defined as unvaccinated and vaccinated, respectively. Participants with uncertain vaccination status were not included in the statistical models. For the unvaccinated population, the distribution of antibody levels was positively skewed and transformed through boxcox transformation (R-package MASS (Modern Applied Statistics with S)) to improve distribution before entry into the model. For the vaccinated population, age distribution was positively skewed and log transformed before entry into the model. The models were fitted in R [25] using the function lmer of the R-package lme4 [26]. We checked for whether the assumptions of normally distributed and homogenous residuals were fulfilled by visually inspecting a qqplot and the residuals plotted against fitted values (both indicated no obvious deviation from these assumptions). We checked for model stability by excluding subjects one at the time from the data which indicated no influential cases to exist. Variance Inflation Factors [27] were derived using the function vif of the R-package car [28] applied to a standard linear model excluding the random effects; there was no indication of collinearity to be an issue. The significance of the full models as compared to the null model (comprising only the random effect) was established using a likelihood ration test (R function anova with argument test set to “Chisq”). To achieve more reliable *p*-values, we fitted the models using Maximum Likelihood (rather than Restricted Maximum Likelihood) [29]. *p*-Values for the individual predictors were based on likelihood ratio tests comparing the full with the respective reduced models (R function drop1) [30]. For comparison of the overall prevalence in the two countries irrespective of vaccination status or other factors Fischer exact test with the significance level set to 0.5 was used.

### 2.6. Longitudinal Study of Unvaccinated People in CIV

To test whether antibody levels changed over time, we used samples collected in CIV in 2006/2007 and 2012. We used a general linear mixed model into which we included collection period (2006/2007 versus 2012) as the fixed effect while controlling for age, gender, and village (random effect). The distribution of antibody levels was positively skewed and transformed through boxcox before entry into the models. Fitting the model and checking the assumptions and stability were done as described above.

## 3. Results

### 3.1. Validation of the In-House ELISA

To enable testing of large serum panels for the serological screening, we established an OPV-specific high-throughput ELISA. To this end, we used whole-cell lysate of VACV-infected cells to include non-structural or non-surface viral proteins in our antigen mix, as previous studies have shown that some of these proteins are highly immunogenic [31,32]. Non-specific antibody binding to non-viral cellular proteins was addressed by parallel testing of binding to non-infected cell lysate.

**Figure 2:**
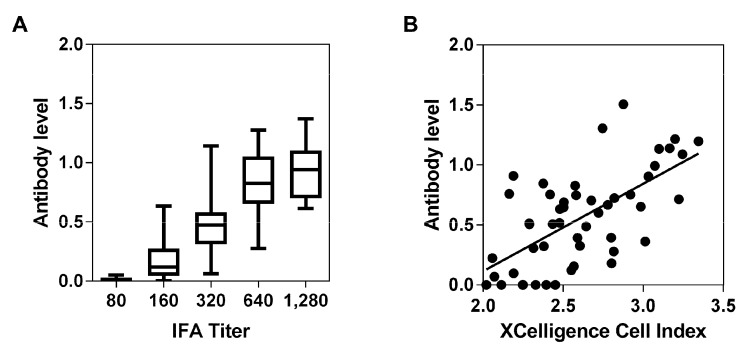
Comparison of ELISA (enzyme linked immunosorbent assay results) (antibody level) and IFA (immunofluorescence assay) titer (**A**) or neutralization assay ((**B**) XCelligence Cell Index) for a panel of sera ((**A**) *n* = 100; (**B**) *n* = 50) collected from German blood donors.

For validation, ELISA results were compared to IFA (*n* = 100) or neutralization test results (*n* = 50) (Figure 2). Here, highly significant correlations (*p* < 0.001) between ELISA and IFA (Spearman *r* = 0.7984) as well as ELISA and neutralization assay (Pearson *r* = 0.6432) indicated a high level of agreement between the newly established ELISA and the well-established IFA as well as a previously established neutralization assay. We therefore used our newly established ELISA to detect the seroprevalence against OPVs.

### 3.2. Serological Screening

**Figure 3:**
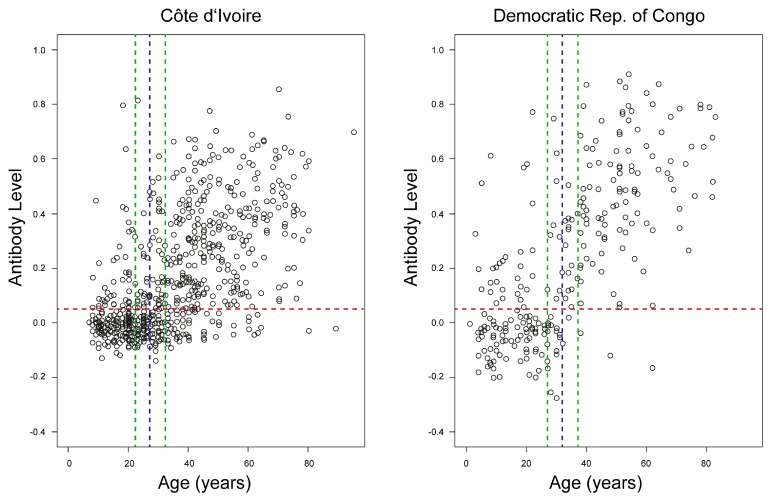
Antibody levels in human participants in CIV and COD. The red horizontal line indicates cut-off value for positive samples. The blue vertical line represents time of vaccination cessation; the green vertical lines indicate the 5-year margins on either side.

The main screening included 737 participants in CIV and 267 participants in COD. In CIV, 379 females and 358 males participated; the median age was 31 years (range 7–95). In COD, 138 females and 129 males participated; the median age was 30 years (range 1–83). The mean number of samples collected per village was 45 (range 9 (Bekombo, COD)–103 (Lompole, COD)). Our ELISA revealed an overall anti-OPV seroprevalence in CIV and COD of 51% (95% CIV 48–55%) and 60% (95% CIV 53–65%), respectively (*p* = 0.015). (Table 1). The antibody levels with respect to age of the participants in the respective countries are shown in Figure 3.

#### 3.2.1. Anti-OPV Seroprevalence in Smallpox-Vaccinated Participants

357 and 112 (total 469) participants were assumed to have been vaccinated in CIV and COD, respectively. Here, the anti-OPV seroprevalence was 80% in CIV (95% confidence interval 75–84%) and 96% in COD (95% confidence interval 91–99%). The antibody levels ranged from −0.166 to 0.912 (mean 0.317). The regression model itself was highly significant (*p* < 0.001) and showed that antibody levels increased with age (*p* < 0.001, mean increase 0.006 pr. year), and were higher in COD than in CIV (*p* < 0.001, mean difference between countries 0.204). There was a small but significant difference between males and females (mean antibody levels of females were 0.043 higher than those of males, *p* = 0.028).

#### 3.2.2. Anti-OPV Seroprevalence in Smallpox Non-Vaccinated Participants

**Figure 4:**
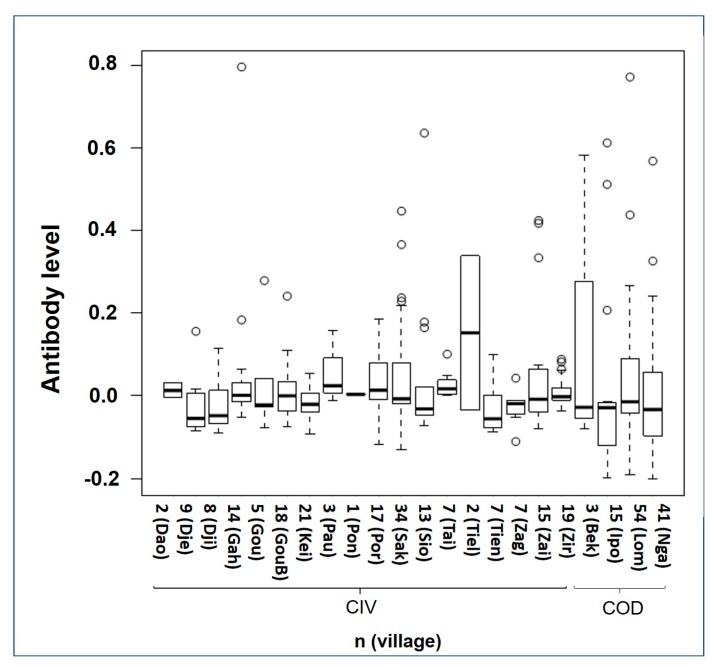
Mean antibody levels in the villages where specimens were taken. The names of the villages are abbreviated to the first three or four letters of the name. The first 18 villages on the left are in CIV, the 4 on the right hand side represents villages in COD.

202 and 113 participants were assumed not to have been vaccinated in CIV and COD, respectively. The OPV seroprevalence was 19% in CIV (95% confidence interval 14–26%) and 26% in COD (95% confidence interval 18–35%). The antibody levels ranged from −0.202 to 0.797 (mean 0.023). Country, gender, and age had no significant effect on antibody levels (*p* = 0.233 for overall model). The median antibody level per village for the unvaccinated population is shown in Figure 4.

Here, no statistical significant differences between the different villages samples were detected regarding antibody level.

#### 3.2.3. Anti-OPV Seroprevalence in Participants with Unclear Smallpox Vaccination Status

178 people in CIV and 42 people in COD had an unclear vaccination status and were excluded from further statistical analyses. The seroprevalence for these participants was 29% (95% confidence interval 23–37) in CIV and 55% (95% confidence interval 39–70) in COD.

#### 3.2.4. Longitudinal Study of Unvaccinated Participants in CIV

In a resampling in 2012, five years after the original sampling in 2006/2007, 75 new specimens from unvaccinated participants were collected in CIV. The OPV seroprevalence in these specimens was 25% (95% confidence interval 16–37%) compared to 19% (95% confidence interval 14–26%) in 2006/2007. However, this increase in anti-OPV-specific antibody levels between the specimens collected from unvaccinated participants in the two sampling periods was not statistically significant controlling for age and gender (mean antibody level 0.055 higher in resampling, *p* = 0.082).

## 4. Discussion

In our study, we assessed the anti-OPV seroprevalence in CIV and COD. The overall anti-OPV seroprevalence was 51% (95% confidence interval 48–55) in CIV and 60% (95% confidence interval 53–65) in COD. As with many comparable studies, our “country level” is based on sampling from selected villages in particular areas and might not be representative of the two countries as a whole, considering that the populations of CIV and COD are approximately 26 and 75 million people, respectively. Moreover, the antibody prevalence might vary even between relatively close geographical areas [33]. Some studies in Ghana and the Republic of Congo report overall anti-OPV seroprevalences of 53% [33] and 57% [34] based on ELISAs, respectively, which is similar to our results. However, other studies employing haemagglutination-inhibition tests describe lower overall seroprevalences in COD (19%, [35]) and Republic of Congo (16%, [36]).

We found that the anti-OPV seroprevalence in smallpox-vaccinated participants in both countries was indeed high with 80% and 96% in CIV and COD, respectively. These results indicate first of all a long-lived immune response after smallpox vaccination and confirm the findings of previous studies [37,38,39]. Both a higher rate of initial vaccination in the COD population as well as repeated contact to circulating OPV might explain the slightly higher overall seroprevalence observed in this population. However, as our presumable vaccination status was based solely on age and we do not have more data on possible OPV exposure we cannot fully explain this observed difference. Our model showed that the antibody levels of vaccinated participants were influenced by country, age, and gender: the level was significantly higher in COD than in CIV, increased with age, and was a slightly higher in women than men. The OPV seroprevalence in smallpox-unvaccinated participants was 19% and 26% in CIV and COD, respectively, and, although we did observe a slight increase, it did not change significantly over time in CIV. These sero-positive, previously unvaccinated people are most likely representative for natural contacts to OPVs. The OPV most prominent in Africa, after the smallpox eradication, is MPXV. According to the literature, there are increasing numbers of MPXV infections in Central Africa, with the COD being a hotspot [4]. Interestingly, we observed no difference in prevalence and antibody level between the CIV and the COD, indicating that the overall exposure to OPVs is the same, and that the relatively higher number of MPXV cases in COD does not make up for a large part of the overall OPV exposure.

While the higher virulence of the Congo clade of MPXV partly explains the larger case numbers observed in COD, a possible explanation for the overall similar seroprevalence of anti-OPV antibodies is that OPVs other than MPXV may be circulating in African countries [40]. Furthermore, the host-range of some OPV is broad and the variety of potential animal reservoirs is extremely high, with many species (such as rodents) living in close contact with humans. Due to the conserved antigenic epitopes among the OPVs, it is not possible to accurately differentiate the immune response to different OPVs with serological techniques [31,41]. Although it seems likely that MPXV contributes to the anti-OPV seroprevalence, at least in endemic regions like the COD, other OPVs should also be considered [33]. Unfortunately, we have no information on suspected skin aberrations of these study participants that could support the seroprevalence data. However, MPXV may also cause subclinical infections which might account for seroconversion in some people [42]. Promising MPXV-specific peptide-based ELISAs might be useful in differentiating between MPXV-specific antibodies and anti-OPV antibodies in the future [43].

As 19% and 26% of unvaccinated participants in CIV and COD are seropositive for OPV, these data indicate at the same time that 81% and 74% of the people who are born after the smallpox vaccination campaign are seronegative for OPV and hence would be sensitive to OPV infections like MPXV. Although the degree of protection afforded by IgG antibodies cannot be determined accurately, the presence of anti-OPV antibodies likely provides some level of protection. It is important to further investigate the long-term dynamics of anti-OPV antibodies and pox disease occurrence, as the population structure will change and the proportion of unvaccinated people will increase. In addition, the age of unvaccinated people will increase and risk factors might become more evident. This effect has already been shown by comparing the age of monkeypox patients in different studies; during surveys in the 1970s and 80s, the majority of the cases were children and the mean age was about 4.5 years. In the surveys performed two decades later, the majority of the patients were older than 25 years and the mean age was approximately fifteen years old [42].

It is not clear why monkeypox occurs more frequently in the Congo Basin than in other African regions. This can only partly be explained by the fact that the Central African strains are more virulent and able to cause human-to-human transmission more efficiently than the West African strains [44,45,46]. Human cases of monkeypox have not been reported in West Africa for decades, although ecological niche models predict areas of suitable ecology in this part of Africa and wildlife studies show that MPXV is indeed present [11,13,16,47].

## 5. Conclusions

Our data show that, even after the eradication of Variola virus, infections with OPVs occur in Africa. These results highlight the need for future monitoring of OPVs in human populations in tropical Africa. Further studies should ideally be multidisciplinary, combining serological monitoring, clinical epidemiology, and human anthropology to complement reservoir, ecology, and climate studies. This way, the disciplines can be synergized to increase our understanding of, and possibly even predict and prevent, spill-over events [16,17,18].

## Figures and Tables

**Table 1:** Anti-OPV seroprevalence in participants living in CIV and COD.

Sample	Côte D’Ivoire		Democratic Rep. of the Congo	
	Prevalence (% (95% CIV))	*n*	Prevalence (% (95% CIV))	*n*
Overall	51 (48–55)	737	60 (53–65)	267
Vaccinated	80 (75–84)	357	96 (91–99)	112
Females	79 (73–85)	180	98 (90–100)	50
Males	80 (74–85)	177	95 (87–98)	62
Unvaccinated	19 (14–26)	202	26 (18–35)	113
Females	15 (9–23)	101	23 (15–34)	69
Males	24 (17–33)	101	30 (18–44)	44
Resampling unvaccinated	25 (16–37)	75	Not done	-
